# Vibrating colon-stimulating capsule to treat chronic constipation: A systematic review

**DOI:** 10.25122/jml-2023-1025

**Published:** 2023-07

**Authors:** Prakash Bruhan Math, Renju Ravi, Tahir Hakami, Saibal Das, Namita Patel

**Affiliations:** 1 Department of Clinical Pharmacology, Faculty of Medicine, Jazan University, Jazan, Saudi Arabia; 2 Indian Council of Medical Research, Centre for Ageing and Mental Health, Kolkata, India; 3 Department of Global Public Health, Karolinska Institutet, Stockholm, Sweden; 4 Department of Preventive and Social Medicine, Jawaharlal Institute of Postgraduate Medical Education and Research, Puducherry, India

**Keywords:** bowel movement, chronic idiopathic constipation, colon, stimulating, vibrating capsule (VC)

## Abstract

In August 2022, the United States Food and Drug Administration issued marketing authorization for an orally administered vibrating colon-stimulating capsule for treating chronic idiopathic constipation. We aimed to review the literature systematically and synthesize evidence on the role of the vibrating capsule in chronic idiopathic constipation. A comprehensive search was conducted on PubMed, Embase, International Clinical Trials Registry Platform (World Health Organization), Cochrane Library databases, and two pre-print servers (medRxiv.org and Research Square) until 31 December 2022, to identify published pre-clinical and clinical original studies evaluating the role of the vibrating capsule in patients with chronic constipation. The studies were critically analyzed, and data were extracted. We identified thirty-three articles and five studies (one pre-clinical, one combined, and three clinical). The pre-clinical studies in dogs revealed no adverse effects of the vibrating capsule. In the clinical studies, there were significant findings observed. The number of spontaneous bowel movements per week and the proportion of patients experiencing an increase of at least one complete spontaneous bowel movement per week were both significantly higher in the group receiving the vibrating capsule compared to the group receiving the sham capsule. No treatment-related serious adverse event was noted. The mild adverse events were vibration sensation, diarrhea, and abdominal discomfort. The efficacy and safety profiles of the vibrating colon-stimulating capsule in treating patients with chronic constipation are promising. However, more robust evidence is required by conducting large randomized clinical trials before conclusively determining its wider use.

## INTRODUCTION

Constipation is defined as the less-frequent passage of stool or the passage of hard stool, characterized by abdominal discomfort and painful defecation [1, 2]. It is often a chronic disorder, especially in the elderly population. Globally, the prevalence of chronic constipation is estimated to be around 14% [3], but it can be higher, reaching up to 22% in elderly individuals and up to 30% in some specific subgroups, such as women, non-whites and those belonging to a lower socioeconomic status [4]. Chronic constipation is classified into idiopathic (normal-transit and slow-transit) and secondary constipation [5]. Chronic constipation adversely impacts direct healthcare expenditures and has a huge negative effect on the working productivity and quality of life of patients [6, 7].

Several therapeutic approaches are available for the treatment of chronic constipation. The World Gastroenterology Organization advocates a staggered approach starting with lifestyle and dietary modifications and escalating to pharmacotherapy (laxatives, prokinetic agents, and enemas). Likewise, the American Society of Colon and Rectal Surgeons recommends plenty of fiber intake, fluid supplementation, and osmotic laxatives as the initial management, followed by stimulant laxatives as the second line of treatment for chronic constipation. Refractory colonic slow-transit constipation may require surgical interventions [1, 5, 8, 9]. While several therapeutic options are available, the persisting high prevalence rate, treatment-related adverse effects, high recurrence rate, and low patient satisfaction level necessitate the discovery of newer and safer approaches for treating chronic constipation.

Several non-pharmacological treatments and novel pharmacological approaches are being explored, such as YKP10811 (5-hydroxytryptamine-4 receptor agonist), relamorelin (ghrelin receptor agonist), and plecanatide (guanylate cyclase C receptor agonist) [10-13]. Additionally, other techniques have been investigated, such as the dispersion of stool facilitating bowel movements by direct mechanical stimulation of the intestine, physical stimulation of the abdomen by abdominal massage [14], whole-body vibration by an external vibrating belt [15, 16], micro-physiotherapy [17], interferential electrical stimulation [18], and acupuncture [19]. These approaches have demonstrated varying results for the treatment of chronic constipation.

The exact mechanism through which physical stimulation helps relieve constipation has not been clearly elucidated. Increased gastrointestinal motility and relaxation of sphincters by parasympathetic and local reflexes might play a role [16]. Vibration may augment the effect by converting external mechanical stimulation into gastrointestinal wall stimulation [20]. In August 2022, the United States Food and Drug Administration (FDA) issued marketing authorization for an orally administered vibrating capsule (VC) for treating chronic idiopathic constipation in patients who have not experienced relief with available laxative therapies for at least a month [21].

**Figure 1:**
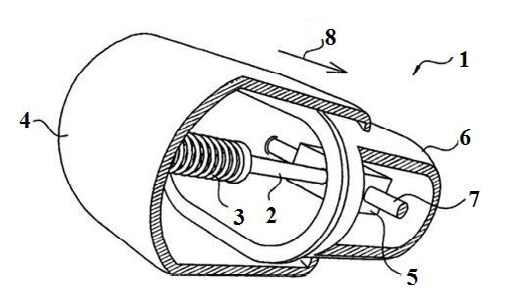
A fragmented isometric view of the vibrating capsule. The shell (1) contains two attached segments that can move independently. One end of a ferromagnetic shaft (2) is connected by a biasing spring (3) to the inner surface of segment 4. The other end of the shaft is free to move axially through the lumen of the solenoid (5), which is connected to the inner surface of segment 6 by a bracket (7). By conducting an electric current through the solenoid, the ferromagnetic shaft and the solenoid are pulled toward each other. When the electric current is stopped, the biasing and connecting springs assume their initial unstressed lengths, while segments 4 and 7 are forced to move in opposite directions toward their initial disposition (8). The figure was reproduced with permission from Ron Y, Halpern Z, Safadi R, Dickman R, et al. Safety and efficacy of the vibrating capsule, an innovative non-pharmacological treatment modality for chronic constipation. Neurogastroenterol Motil Off J Eur Gastrointest Motil Soc. 2015;27:99–104.

The VC system is made up of a single-use capsule and a control pod controlling that activates the capsule using an electromagnetic signal. The vibrating sequence initiates after a programmed delay of 6–8 hours to allow the capsule to enter the colon. The vibration mode can be adjusted and modulated using an external configurator operated through a smartphone application (Figure 1). Previous studies have shown that the VC system has the potential to significantly increase the frequency of bowel movements [22-24]. We aimed to review the existing literature and synthesize pre-clinical and clinical evidence regarding the efficacy and safety of VC in the treatment of chronic idiopathic constipation.

## MATERIAL AND METHODS

### Eligibility criteria

We aimed to include all completed and published original pre-clinical and clinical interventional studies in the English language that evaluated the role of vibrating colon-stimulating capsules in adult patients (≥18 years) with chronic idiopathic constipation. Case reports, case series, commentaries, reviews, viewpoints, editorials, or opinions were excluded.

### Search strategy

We searched PubMed, Embase, International Clinical Trials Registry Platform (World Health Organization), Cochrane Library databases [Cochrane Database of Systematic Reviews, Cochrane Central Register of Controlled Trials (CENTRAL), and Cochrane Methodology Register], and two pre-print servers (medRxiv.org and Research Square) from inception until 31 December 2022. The search terms used in various combinations were “constipation”, “chronic constipation”, “chronic idiopathic constipation”, “functional constipation”, “capsule”, “vibrating”, “vibration”, “vibrating capsule”, “vibrant”, “colon”, “colon-stimulating”, “large intestine”, “large intestine-stimulating”, and “stimulating”. The search strings were modified for different bibliographic databases in combination with database-specific filters. The titles and abstracts of the relevant articles in English were obtained using the search strategy. Two authors independently screened the articles, retrieved abstracts, and obtained the full text of selected articles when necessary. In case of disagreements, a resolution was reached through discussion involving a third author.

### Analysis of the selected articles

We critically analyzed all the included studies. The risk of bias of each study was analyzed using the Systematic Review Centre for Laboratory Animal Experimentation (SYRCLE) tool for pre-clinical studies, the Risk of Bias in Non-randomized Studies of Intervention (ROBINS-I) tool for the non-randomized study, and the revised Cochrane risk-of-bias 2 tools for randomized controlled trials. We extracted data related to the key efficacy and safety outcomes of the vibrating capsule from the included studies. We did not make any assumptions or simplifications during the process. Descriptive statistics were used, and no pooled analysis was performed.

## RESULTS

The literature search yielded 33 potential studies, of which six studies [22, 23, 25-27] were included in our review. The study flow chart depicting the steps of the synthesis of evidence from the literature is illustrated in Supplementary Figure s1. The risk of bias in the studies was moderate (Supplementary Table s1).

### Results of pre-clinical studies

In the first phase of a study by Ron *et al*., three dogs were administered a sham capsule followed by 13 vibrating capsules (five for two dogs and three for one). The dogs were followed up for 90 days. The behavior of all dogs was normal, without any sign of discomfort, and there was no difficulty in expulsing the capsules. In the second phase, two dogs were each administered two sham capsules and two vibrating capsules. The first dog had no difficulty expelling the capsules, and no discomfort was noted. The second dog showed some discomfort, and no bowel movement could be recorded for three days following the sham capsule administration. However, by the end of six days, the dogs expelled all capsules and showed no additional signs of discomfort. There were no visible signs of injury observed in any of the dogs [22].

In the study by Yu *et al*., eight beagle dogs were administered a vibrating capsule coupled with a smartphone-controlled external configurator. In the ultra-high frequency mode, the capsules were located in the rectum after vibrating for 3–4 h, significantly reducing the expulsion time compared to the high-frequency mode. No adverse effect was noted, and there were no signs of perforation, ulceration, or macroscopic bleeding from the gastrointestinal tract. In terms of efficacy, the defecation frequency increased after the administration of the capsule without affecting the stool characteristics. In addition, the time of capsule evacuation was reduced without any significant difference across different modes (Table 1) [25].

**Table 1:** Summary of pre-clinical study results for vibrating capsule as a treatment for chronic constipation

Author, year, country	Sample size	Animal used	Primary outcome	Main efficacy results	Main safety results
Ron *et al*., 2015, Israel [22]	5	Dogs	Safety parameters		No adverse effect was noted No problem with capsule expulsion
Yu *et al*., 2017, China [25]	8	Beagle dogs	Safety parameters	Defecation frequency increased without influencing stool characteristics	No adverse effect was noted

### Results of clinical studies

Table 2 displays the key findings and characteristics of clinical studies evaluating the effect of VC on chronic constipation treatment. In phase 1 of a study by Ron *et al*. (n=6 patients; age range: 21–41 years; females: 50%), the mean time for capsule expulsion was 2.2 days, with no abnormalities seen in vital signs, blood investigations, and electrocardiogram up to seven days. In the second phase, a non-randomized, single-group study was conducted among 26 patients (age: 47±12.6 years; females: 89.3%) with constipation, who responded unsatisfactorily to other available treatments. A significant increase in the mean number of spontaneous bowel movements per week (1.60±1.09 [2.19±0.67 to 3.79±1.31], p<0.001) was noted with VC, with an 88.5% responder rate (defined as an increase of at least one complete spontaneous bowel movement per week compared to the baseline). All patients expelled the capsule without complications. However, 27 transient non-serious adverse events, primarily abdominal pain, diarrhea, and flatulence, were reported by 12 patients [22].

**Table 2:** Summary of clinical study results of vibrating capsule for treating chronic constipation

Author, year, country	Sample size	Age, gender	Study population	Interventional arm (dose, duration)	Comparator arm (dose, duration)	Primary outcome	Main efficacy outcomes	Main safety outcomes
Ron *et al*., 2015, Israel [22]	6	21–41 years, 50% females	Healthy volunteers (safety study) and patients with constipation (efficacy study)	Safety study: single vibrating capsule Efficacy study: vibrating capsule twice a week for 7.5 weeks	Sham capsule (same dose and duration)	Safety parameters	Mean time to expulsion was 2.2 days Significant increase of 1.60±1.09 (2.19±0.67 to 3.79±1.31, p<0.001) in the mean number of spontaneous bowel movements per week	Non-serious adverse events were reported by 12 out of 26 patients
Nelson *et al*., 2017, United States of America [26]	24	B44.2±8.1 years, 100% females	Patients with functional constipation (Rome III criteria)	Vibrating capsule twice a week for 8 weeks	Sham capsule (same dose and duration)	Colonic geometric center at 48 h Half-life of the capsule in the ascending colon	No significant differences in the overall colonic transit between the vibrating capsule and the sham capsule groups until 48 h [colonic geometric center at 48 h, 3.46 (IQR, 2.55–4.61) vs. 2.76 (IQR, 2.42–4.03) (p=0.13)] Predicted numerical increase in the predicted Bristol stool form in the sham capsule group [2.9 (IQR, 2.7–3.7)] as compared to the vibrating capsule group [3.3 (IQR, 2.7–4.2)] No significant difference in the half-life of the capsule in the ascending colon between the vibrating capsule and the sham capsule groups [19.2 h (IQR, 13.03–21.39) vs. 12.6 h (IQR, 7.12–17.66)]	
Rao *et al*., 2020, United States of America [23]	250	Mean age, 41–45 years; 78% of females	Patients with chronic idiopathic constipation	First phase: vibrating capsule twice a week for 8 weeks Second phase: vibrating capsule (two different modes) five times a week for 4 weeks and then twice a week for another 4 weeks	Sham capsule (same dose and duration)	Responder rate (an increase of at least one complete spontaneous bowel movement per week as compared to the baseline)	In the first phase, 50% of complete spontaneous bowel movements were reported in the vibrating capsule group as compared to 42% in the sham capsule group (p<0.01) Responder rate did not differ between the vibrating capsule and sham capsule groups (27.9% vs. 35.9%, p=0.19) In the second phase, 21.5% of complete spontaneous bowel movements were reported in the vibrating capsule group (mode 1) as compared to 11.5% in the sham capsule group (p=0.04) Responder rate did not differ between the vibrating capsule and sham capsule groups (mode 1, 50% vs. 31.8%, p=0.24 and mode 2, 36.4% vs. 31.8%, p=0.75). A higher frequency of complete spontaneous bowel movements was observed in the combined vibrating capsule groups as compared to the combined sham capsule groups	One unrelated serious adverse event in each group (first phase) and no serious adverse event (second phase) were noted Mild adverse events were vibration sensation and diarrhea
Zhu *et al*., 2022, China [27]	106	Mean age, 42.8–43.2 years; 91% of females	Patients with functional constipation (Rome IV criteria)	Vibrating capsule twice a week for 6 weeks	Sham capsule (same dose and duration)	Responder rate (an increase of at least one complete spontaneous bowel movement per week as compared to the baseline)	Responder rate in the vibrating capsule group was significantly higher than that in the sham capsule group (64.2% vs. 35.8%, p<0.01) Change in the median weekly complete spontaneous bowel movements from baseline was significantly greater in the vibrating capsule group than in the sham capsule group during the first two weeks (difference, 0.50; 95% CI, 0 to 1.18; p=0.02) and the entire treatment period (difference, 0.52; 95% CI, 0.02 to 1.03; p=0.02) Mean Patient Assessment of Constipation-Symptoms score and Patient Assessment of Constipation-Quality of Life score differed significantly from the baseline in both groups (all p<0.001) Mean expulsion time did not differ significantly between the vibrating capsule and sham capsule groups (52.78 h vs. 54.93 h, p=0.77).	No treatment-related serious adverse event was noted Mild adverse event was abdominal discomfort.

In a small randomized controlled trial (RCT) by Nelson *et al*. (n=24 patients; age: 44.2±8.1 years; females: 100%), patients with functional constipation (Rome III criteria) were randomized to VC (n=12) or sham capsule (n=12), twice a week for eight weeks. No significant differences were observed in the overall colonic transit time between the VC and sham capsule groups within 48 hours [colonic geometric center at 48 hours, 3.46 (IQR, 2.55–4.61) *vs*. 2.76 (IQR, 2.42–4.03) (p=0.13)]. In addition, there was no significant difference in the half-life of the capsule in the ascending colon between the VC and sham capsule groups (19.2 h [IQR, 13.03–21.39] *vs*. 12.6 h [IQR, 7.12–17.66]). Interestingly, there was a predicted numerical increase in the Bristol stool scale (looser consistency) in the sham capsule group [2.9 (IQR, 2.7–3.7)] compared to the VC group [3.3 (IQR, 2.7–4.2)]. Safety parameters were not reported in this study [26].

In the first phase of an RCT by Rao *et al*. (n=182 patients; mean age: 41–45 years; females: 78%), patients with chronic idiopathic constipation were randomized to a VC (n=89) or sham capsule (n=93) five times a week for eight weeks. A higher proportion of complete spontaneous bowel movements was found either during the vibration session or up to three hours after the session. Patients in the VC group reported a significantly greater number of complete spontaneous bowel movements during or near the vibration time than those in the sham capsule group (50% *vs*. 42%; p<0.01). However, the responder rate did not differ between the VC and sham capsule groups (27.9% *vs*. 35.9%, p=0.19). Each group had one serious adverse event (anxiety attack); however, both were unrelated to the study medications. In the second phase of the trial, after a run-in period of two weeks, 68 patients (mean age: 41–45 years; females: 78%) with chronic idiopathic constipation were randomized to a VC or sham capsule (n=24) five times a week for four weeks and then twice a week for another four weeks. A significantly greater number of complete spontaneous bowel movements were reported by patients in the VC group compared with those in the sham capsule group (21.5% *vs*. 11.5%; p=0.04). However, the responder rate did not differ between the VC and sham capsule groups (mode 1, 50% *vs*. 31.8%, p=0.24 and mode 2, 36.4% *vs*. 31.8%, p=0.75). No serious adverse events were reported. In the pooled analysis, a higher frequency of complete spontaneous bowel movements was noted in the combined VC groups compared with the combined sham capsule groups. An additional peak indicating a residual effect of the capsule after the last intake was also noted. The overall proportions of mild adverse events (mainly vibration sensation and diarrhea) were low in both groups [23].

In another RCT by Zhu *et al*. (n=106 patients; mean age: 42.8–43.2 years; females: 91%), patients with functional constipation (Rome IV criteria) were randomized to a VC (n=53) or sham capsule (n=53) twice a week for six weeks. The responder rate in the VC group was significantly higher compared to the sham capsule group (64.2% *vs*. 35.8%, p<0.01). More patients in the VC group reported at least one weekly spontaneous bowel movement at week four of treatment (difference, 22.7%; 95% CI: 8 to 46; p=0.02) and follow-up (difference, 17.3%; 95% CI, 0 to 35; p=0.04) periods. The change in the median weekly complete spontaneous bowel movements from baseline was significantly greater in the VC group compared to the sham capsule group during the first two weeks (difference, 0.50; 95% CI, 0.00 to 1.18; p=0.02) and the entire treatment period (difference, 0.52; 95% CI, 0.02 to 1.03; p=0.02). More patients in the VC group reported at least three weekly spontaneous bowel movements at week six (difference, 11%; 95% CI: −8 to 30; p=0.27). In addition, there were significant differences in the mean change in the Patient Assessment of Constipation-Symptoms score and Patient Assessment of Constipation-Quality of Life score from baseline in both groups (p<0.001). However, the administration of VC did not significantly affect stool consistency. In addition, the mean expulsion time did not significantly differ between the VC and sham capsule groups (52.78 hours *vs*. 54.93 hours, p=0.77). No treatment-related serious adverse events occurred, and abdominal discomfort (3.7%) was the most common adverse event associated with VC [27].

## DISCUSSION

To the best of our knowledge, this is the first comprehensive review highlighting the efficacy and safety profile of VC for the treatment of chronic idiopathic constipation. Pre-clinical studies have demonstrated the safety of VC. Evidence from clinical studies suggests that VC can increase the number of complete spontaneous bowel movements per week in patients with chronic idiopathic constipation. No treatment-related serious adverse events were noted, and the mild adverse events observed were vibration sensation, diarrhea, and abdominal discomfort. VC can potentially augment bowel movements, promote defecation, ameliorate symptoms, and improve the quality of life in patients with chronic constipation [22, 25].

Although several treatment modalities exist for chronic constipation, they may occasionally cause unpredictable bowel movements leading to diarrhea [27, 28]. An internet survey by Müller-Lissner *et al*. found that the degree of satisfaction in patients using existing therapeutic options for chronic constipation was low, thereby suggesting the need for more innovative and safer therapeutic strategies [29]. Currently, reducing the symptom severity, prolonging the therapeutic effect of medications, and improving patients' quality of life are the main treatment targets in patients with chronic constipation. The favorable treatment effects and safety profile of VC make it a promising therapeutic option for patients with chronic idiopathic constipation. VC promotes defecation by directly stimulating the colonic walls and inducing bowel movements [20]. However, more reliable evidence from larger RCTs over longer follow-up durations is required to confirm the existing findings.

VC also enhances the normal physiological effects of waking up and food intake on bowel movements [22-24]. The biological circadian rhythm leads to a significant reduction in the colonic pressure activity at night and a marked increase in the pressure activity after waking up or after food intake [30]. The gastrointestinal tract has an internal clock that responds to the body's circadian rhythm. However, it also functions independently to some extent and has an intrinsic ability to respond to various environmental stimuli, including alterations in eating schedules and habits [31]. Biological clock genes, particularly the period circadian clock 2 (Per2) gene, are expressed within the epithelial cells and myenteric plexus of the colon and demonstrate circadian rhythm [32]. Patients with chronic slow transit constipation have significantly reduced colonic pressure activity after waking up or food intake compared to healthy individuals [33]. A study by Shemerovskii *et al*. found that healthy individuals have daily bowel movements in the morning between 6 am and noon, while those with irregular bowel habits have daily bowel movements only three to four times a week, predominantly between 8 pm and midnight [34]. Hence, the human colon has been naturally programmed to empty in the morning after awakening, and this biorhythm can potentially be altered by VC to achieve beneficial treatment effects in patients with chronic constipation [35].

However, certain issues related to VC need to be addressed. First, a detailed understanding of its mechanism of action and modes of operation, including vibration parameters (onset duration, frequency, and amplitude), is required. Data regarding the successful triggering of the frequency or amplitude of intestinal contractions is limited. The lack of response in some patients might be due to unknown biological factors having inter-individual variations, such as abnormalities in afferent or efferent physiological mechanisms that are required to respond to the vibrating stimulus. Furthermore, a vast majority of patients with constipation unassociated with dyssynergia have a normal colonic transit. It is imperative to evaluate the efficacy and safety of VC in these subgroups of patients [25]. The optimal timing of capsule administration for obtaining maximum benefit also needs to be ascertained. In addition, the effect of VC on patients with secondary constipation needs to be evaluated. Finally, the risks of capsule retention should be explored as well [36].

Certain limitations must be kept in mind while interpreting our review. First, the sample sizes of the included studies were small. However, results from ongoing well-powered clinical trials will enable definite conclusions to be reached in the future. Secondly, data regarding the frequency and duration of VC varied across the included studies. In addition, the duration of treatment was short (mostly eight weeks); hence, the long-term efficacy and safety of VC could not be determined. Furthermore, our review mainly focused on the adult Caucasian and Chinese populations. Hence, the generalizability of these findings over other populations with different dietary habits and medical comorbidities requiring multiple medications is questionable.

## CONCLUSION

The efficacy and safety profiles of VC in treating patients with chronic idiopathic constipation are promising. VC can potentially increase the number of complete spontaneous bowel movements per week in patients with chronic idiopathic constipation, with only mild adverse events like vibration sensation, diarrhea, and abdominal discomfort and no treatment-related serious adverse effects. However, more reliable evidence from large RCTs over longer follow-up durations is required to reach definite conclusions regarding the feasibility of its usage for the treatment of chronic idiopathic constipation.

## Supplementary Material



## Data Availability

The datasets generated and/or analyzed during the current study are available from the corresponding author upon reasonable request.
