# Supplementary material for: Vibrating colon-stimulating capsule to treat chronic constipation: A systematic review
Source: J Med Life. 2023 Jul;16(7):1050–6. doi: 10.25122/jml-2023-1025 (PMC10600682; doi:10.25122/jml-2023-1025)
Supplement: Supplementary file 1 [file JMedLife-16-1050-s001.pdf]

Supplementary Material

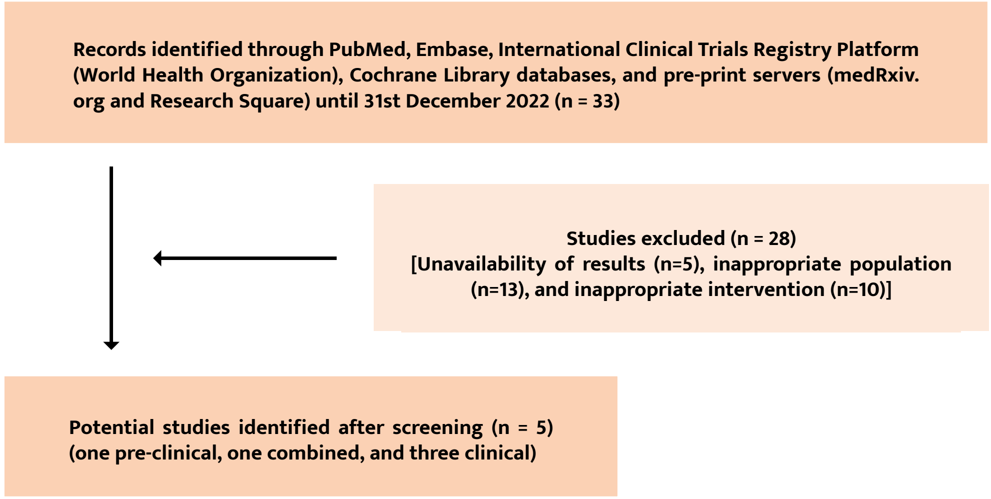

Supplementary Figure s1. Flowchart depicting the steps of evidence synthesis from literature

Supplementary Table s1. Risk of bias in the studies included

Tool	Parameters	Studies				
		Yu, et al., 2017 [25]	Ron, et al., 2015 [26]	Nelson, et al., 2017 [27]	Rao, et al., 2020 [28]	Zhu, et al., 2022 [29]
Systematic Review Centre for Laboratory animal experimentation (SYRCLE) tool [22]	Selection bias (sequence generation, baseline characteristics, allocation concealment)	Moderate	Moderate			
	Performance bias (random housing, blinding)	Low	Low			
	Detection bias (random outcome assessment, blinding)	Low	Low			
	Attrition bias (incomplete outcome data)	Low	Low			
	Reporting bias (Selective outcome reporting)	Low	Low			
	Other bias	Moderate	Moderate			
Risk of Bias in Non-randomized Studies - of Intervention (ROBINS-I) tool [23]	Bias due to confounding		Moderate			
	Bias in selection of participants for the study		Low			
	Bias in classification of interventions		Low			
	Bias due to deviations from intended interventions		Low			
	Bias due to missing data		Moderate			
	Bias in measurement of outcomes		Low			
	Bias in selection of the reported result		Low			
	Overall bias		Moderate			
Revised Cochrane risk-of-bias 2 tools for randomized controlled trials [24]	Bias arising from the randomization process			Low	Low	Low
	Bias due to deviations from intended interventions			Low	Low	Low
	Bias due to missing outcome data			Low	Low	Low
	Bias due to measurement of the outcome			Moderate	Moderate	Moderate
	Bias in the selection of the reported result			Low	Low	Low
	Overall bias			Moderate	Moderate	Moderate
